# High dose of dexamethasone attenuates docetaxel-induced fluid retention in breast cancer treatment

**DOI:** 10.1038/s41598-023-36264-4

**Published:** 2023-06-07

**Authors:** Yoshitaka Saito, Ryota Kanno, Yoh Takekuma, Takashi Takeshita, Tomohiro Oshino, Mitsuru Sugawara

**Affiliations:** 1 Department of Pharmacy, Hokkaido University Hospital, Kita 14-jo, Nishi 5-chome, Kita-ku, Sapporo, 060-8648 Japan; 2 Department of Breast Surgery, Hokkaido University Hospital, Kita 14-jo, Nishi 5-chome, Kita-ku, Sapporo, 060-8648 Japan; 3 Laboratory of Pharmacokinetics, Faculty of Pharmaceutical Sciences, Hokkaido University, Kita 12-jo, Nishi 6-chome, Kita-ku, Sapporo, 060-0812 Japan

**Keywords:** Breast cancer, Cancer therapy

## Abstract

Docetaxel-induced fluid retention (DIFR) cumulatively occurs and is one of the most troublesome adverse effects. This study aimed to determine whether high dose dexamethasone (DEX) could prevent DIFR during breast cancer treatment. Breast cancer patients receiving docetaxel (75 mg/m^2^)-containing regimens were divided into 4 and 8 mg/day DEX groups, with each DEX dose administered on days 2–4 and retrospectively assessed. Incidence of greater than or equal to grade 2 DIFR was significantly lower in the 8 mg group (13.0%) compared to the 4 mg group (39.6%, *P* = 0.001). All-grade DIFR was also less in the 8 mg group (*P* = 0.01). Furthermore, the maximum variation of body weight was significantly lower in the 8 mg group (*P* = 0.0003). These results were also confirmed in the propensity score-matched population. Additionally, time-related DIFR incidence was also significantly delayed in the 8 mg group (*P* = 0.0005). Our study revealed that high dose DEX prevents DIFR. Therefore, further studies on its management are required for less onerous chemotherapy provision with better DIFR control.

## Introduction

Docetaxel is one of the most effective chemotherapeutic agents in perioperative and advanced breast cancer treatment^1–3^. In contrast, its administration induces severe neutropenia, peripheral neuropathy, stomatitis, pain, skin toxicity, and fluid retention^1–3^. Among the adverse effects, fluid retention, particularly peripheral edema, is well known. Docetaxel-induced fluid retention (DIFR) cumulatively occurs in 44–65% of docetaxel-administered patients^4^, and the incidence notably increases when its cumulative dosage exceeds 400 mg/m^2^^5^. Severe symptom significantly reduces the patient’s quality of life (QOL) and activities of daily living (ADL). Capillary hyperpermeability is the major mechanism underlying DIFR^6,7^.

Management of DIFR is prevention using corticosteroids^8^ and symptom control with diuretics^4^. Piccart et al. reported that patients who received 40 mg methylprednisolone for three days, from day − 1 to 1, and 7 to 9, for docetaxel on day 1 and 8, had a delayed onset of DIFR compared to non-administered patients (median time to onset: 84 vs. 62 days) and obtained a higher median cumulative dose of docetaxel before symptom onset (333 vs. 215 mg/m^2^)^8^. However, dexamethasone (DEX) 8–16 mg/day for 3–5 days was administered for DIFR prophylaxis in other clinical studies^2,4,9^, suggesting that the appropriate dosage of corticosteroids for DIFR prevention remains unclear. At the Hokkaido University Hospital, prophylactic corticosteroids were administered as oral DEX 4 mg once a day on days 2–4. This dosage was changed to 8 mg orally on days 2–4, according to previous reports^2,4,9^. Therefore, this study aimed to determine the dose-dependent preventive effects of DEX on DIFR.

## Results

### Patient characteristics

**Figure 1:**
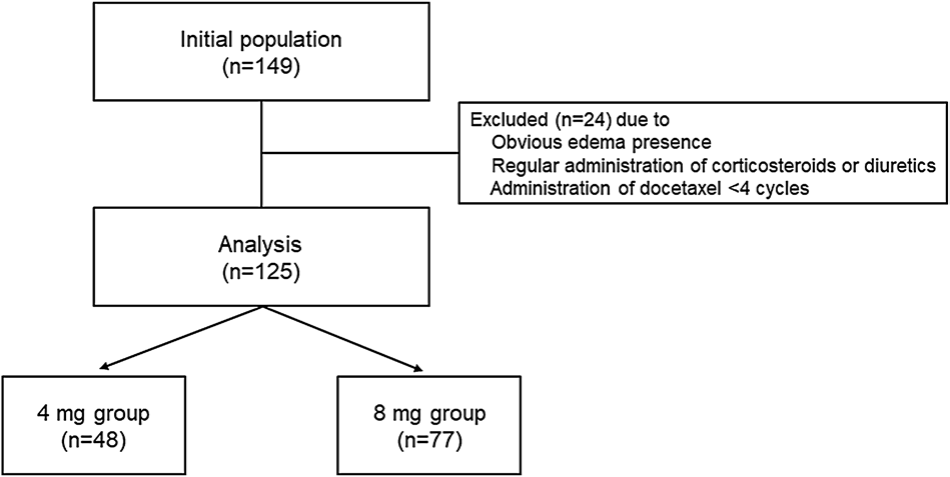
Study design.

In total, 125 patients were enrolled in this retrospective observational study (Fig. 1). Baseline patient characteristics are shown in Table 1. In the all-patient population, there were no significant differences between the two groups in sex, Eastern Cooperative Oncology Group performance status (ECOG-PS), prior treatment history, hormonal receptor expression, human epidermal growth factor receptor-2 (HER2) overexpression, Ki-67, lymph node dissection, body surface area (BSA), body mass index (BMI), liver dysfunction (grade 1 or higher aspartate aminotransferase, alanine aminotransferase, total bilirubin elevation), serum albumin, regular alcohol intake (≥ 5 days in a week), smoking history at baseline, and co-administration of pegfilgrastim. In contrast, patients in the 8 mg DEX group were significantly older and in a more advanced stage and had lower creatinine clearance (CCr). However, no background differences were observed between the groups in the propensity score-matched population. There were no patients with prior radiotherapy or cardiovascular disease other than hypertension in this study.

**Table 1:** Patient characteristics.

	All-patient analysis			Propensity-matched analysis		
	4 mg group	8 mg group	*P* value	4 mg group	8 mg group	*P* value
No. of patients	48	77		39	39	
Sex (male/female)	1/47	2/75	1.00	0/39	0/39	1.00
Age (median, range)	50 (30–67)	56 (27–73)	0.02*	55 (32–67)	53 (27–73)	0.83
Performance status (n)						
0–1	48	77	1.00	39	39	1.00
**Staging (n, %)**						
1–3	47 (97.9%)	67 (87.0%)		38 (97.4%)	39 (100%)	
4 or recurrence	1 (2.1%)	10 (13.0%)	0.049*	1 (2.6%)	0 (0%)	1.00
Prior treatment history (n, %)	36 (75.0%)	60 (77.9%)	0.83	31 (79.5%)	32 (82.1%)	1.00
Histology						
ER-positive, PR-positive, or both (n, %)	28 (58.3%)	41 (53.3%)	0.71	19 (48.7%)	20 (51.3%)	1.00
HER2 overexpression (n, %)	14 (29.2%)	27 (35.1%)	0.56	12 (30.8%)	11 (28.2%)	1.00
Ki-67 (%) (median, range)	49.1 (1.3–97.6)	42.7 (1.6–95.9)	0.47	50.1 (1.3–97.6)	42.7 (4.7–95.9)	0.57
Lymph node dissection (n, %)	8 (16.7%)	15 (19.5%)	0.81	6 (15.4%)	6 (15.4%)	1.00
BSA (m^2^) (median, range)	1.55 (1.40–1.94)	1.54 (1.30–2.00)	0.41	1.53 (1.40–1.94)	1.51 (1.30–1.92)	0.40
BMI (kg/m^2^) (median, range)	22.4 (17.9–36.5)	23.2 (16.3–36.6)	0.53	22.1 (17.9–36.5)	22.2 (16.3–36.6)	0.91
Liver dysfunction (n, %)	20 (41.7%)	24 (31.2%)	0.25	16 (41.0%)	13 (33.3%)	0.64
CCr (mL/min) (median, range)	100.7 (70.6–211.4)	91.7 (59.5–223.2)	0.001**	95.0 (70.6–168.3)	95.1 (60.9–223.2)	0.24
Serum albumin (g/dL) (median, range)	4.1 (3.6–5.0)	4.0 (3.2–4.9)	0.08	4.0 (3.6–5.0)	4.1 (3.4–4.9)	0.64
Alcohol intake (≥ 5 days in a week) (n, %)	10 (20.8%)	9 (11.7%)	0.20	6 (15.4%)	7 (18.0%)	1.00
Smoking history (former or current) (n, %)	23 (47.9%)	40 (52.0%)	0.71	19 (48.7%)	16 (41.0%)	0.65
Current smoker	6 (12.5%)	14 (18.2%)	0.46	4 (10.3%)	5 (11.8%)	1.00
Co-administration of pegfilgrastim (n, %)	4 (8.3%)	14 (18.2%)	0.19	3 (7.7%)	2 (5.1%)	1.00
Treatment regimen (n, %)						
Docetaxel	24 (50.0%)	43 (55.8%)		20 (51.3%)	25 (64.1%)	
Docetaxel + trastuzumab	14 (29.2%)	8 (10.4%)		12 (30.8%)	4 (10.3%)	
Docetaxel + cyclophosphamide	10 (20.8%)	7 (9.1%)		7 (18.0%)	3 (7.7%)	
Docetaxel + trastuzumab + pertuzumab	0 (0%)	19 (24.7%)		0 (0%)	7 (18.0%)	

Liver dysfunction: grade 1 or higher aspartate aminotransferase, alanine aminotransferase, total bilirubin elevation.

*ER* estrogen receptor, *PR* progesterone receptor, *HER2* human epidermal growth factor receptor 2, *BSA* body surface area, *BMI* body mass index, *CCr* creatinine clearance.

*P < 0.05; **P < 0.01.

### Evaluation of DIFR incidence

**Figure 2:**
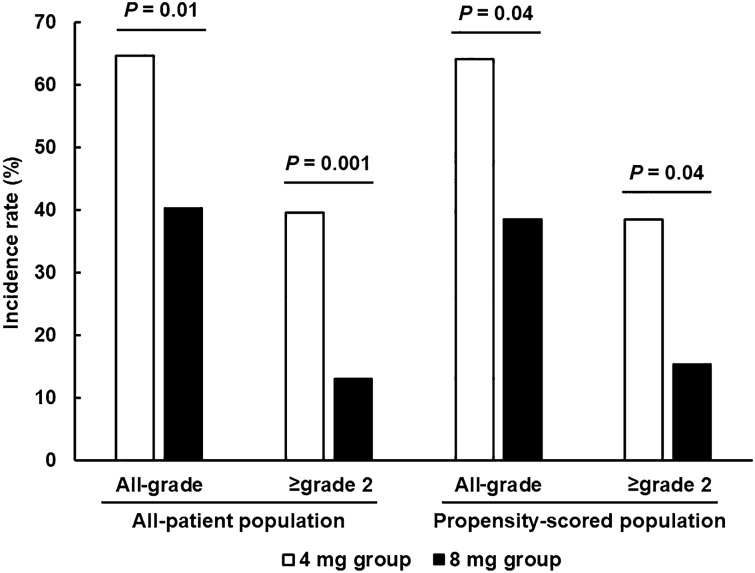
Incidence of DIFR.

The incidence of DIFR is shown in Fig. 2. The incidence of greater than or equal to grade 2 DIFR, which is a primary symptom of this study, was significantly lower in the 8 mg group (13.0%) compared to the 4 mg group (39.6%), which met the primary endpoint of this study (*P* = 0.001). The incidence of all-grade DIFR was 64.6% in the 4 mg group and 40.3% in the 8 mg group, which was also significantly lowered by the DEX dose increase (*P* = 0.01). These results were also confirmed in the propensity score-matched population. Maximum variation of body weight was 2.47 ± 2.00 kg and 1.15 ± 1.82 kg in the 4 and 8 mg groups in the all-patient population, respectively, which was significantly lower in the 8 mg group (*P* = 0.0003) with similar results in the propensity score-matched population (data not shown). In contrast, the incidence of grade 3 DIFR was 4.2% in the 4 mg group and 1.3% in the 8 mg group among the all-patient population (*P* = 0.56) and 5.1% and 2.6% in the propensity score-matched population (*P* = 1.00), respectively, which was not different.

**Figure 3:**
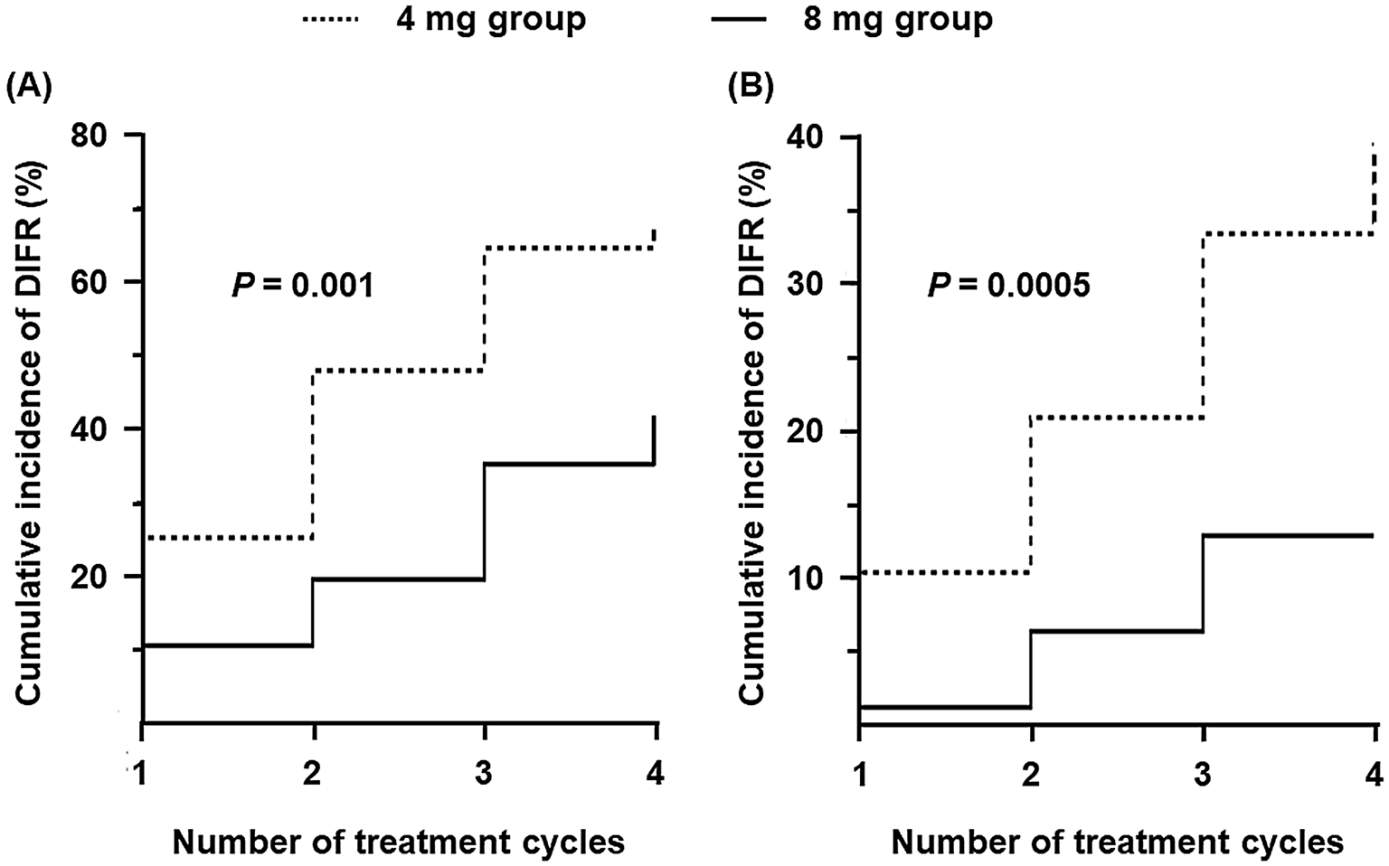
Onset time of (**A**) all-grade and (**B**) greater than or equal to grade 2 DIFR between groups in the all-patient population.

**Figure 4:**
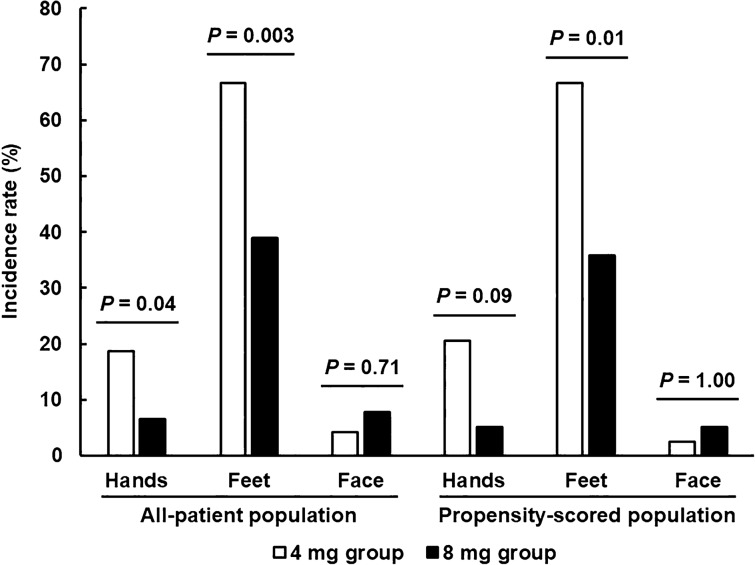
Incidence of DIFR at each site between the groups.

The time-related DIFR incidence in the all-patient population was also evaluated, resulting in a significant delay in the 8 mg group (*P* = 0.001 and 0.0005 for all-grades and greater than or equal to grade 2 DIFR, respectively; Fig. 3). Additionally, we also evaluated the sites of edema. Edema in the hands and feet was significantly less in the 8 mg group, whereas facial edema similarly appeared in the all-patient population (Fig. 4). These results were also confirmed in the propensity score-matched population, except for the edema in the hands.

### Risk analysis for DIFR development

Multivariate logistic regression analyses have shown that 8 mg/day DEX administration is a singular factor associated with greater than or equal to grade 2 DIFR reduction (adjusted odds ratio 0.20; 95% confidence interval [95% CI] 0.08–0.49, *P* = 0.0005, Table 2A). Moreover, it was also suggested as an independent factor for all-grade DIFR reduction (0.35; 0.16–0.77, *P* = 0.009, Table 2B). In contrast, patients with prior treatment history were at higher risk for all-grade DIFR development (2.47; 1.00–6.10, P = 0.049, Table 2B). Smoking history tended to worsen the incidence of greater than or equal to grade 2 DIFR, but it was not significant. Patients in the 8 mg group tended to receive more dose reduction compared to the 4 mg group in the all-patient population, which could have influenced the obtained results (11.7 vs. 2.1%, *P* = 0.09, up to 20% reduction). However, in the analysis, we were unable to evaluate the association between dose reduction during the treatment and greater than or equal to grade 2 DIFR development, as no patient with reduction experienced the symptom.

**Table 2:** Univariate and multivariate analyses for risk factors associated with (A) greater than or equal to grade 2 and (B) all-grade DIFR.

	Univariate analysis		Multivariate analysis	
	Odds ratio (95% CI)	*P* value	Odds ratio (95% CI)	*P* value
**(A)**				
*Age (years)*				
≥ 65/ < 65	0.74 (0.23–2.42)	0.20	Excluded	–
*Sex*				
Female/male	0.60 (0.05–6.82)	0.68	Excluded	–
*Staging*				
Metastatic/others	0.31 (0.04–2.51)	0.27	Excluded	–
*Prior treatment history*				
Present/absent	2.20 (0.70–6.95)	0.18	2.82 (0.83–9.67)	0.10
*Hormonal receptors*				
ER, PR-positive or both/Negative	1.44 (0.62–3.38)	0.40	Excluded	–
*HER2 overexpression*				
Positive/negative	1.10 (0.46–2.65)	0.83	Excluded	–
*Lymph node dissection*				
Present/absent	0.65 (0.20–2.09)	0.47	Excluded	–
*BSA (m*^*2*^*)*				
≥ 1.5/ < 1.5	0.78 (0.33–1.85)	0.57	Excluded	–
*BMI (kg/m*^*2*^*)*				
≥ 25/ < 25	0.58 (0.22–1.50)	0.26	Excluded	–
*Liver dysfunction*				
Present/absent	0.96 (0.40–2.30)	0.93	Excluded	–
*Hypoalbuminemia*				
Present/absent	1.16 (0.51–2.67)	0.72	Excluded	–
*Alcohol intake (*≥ *5 days in a week)*				
Yes/no	1.22 (0.40–3.73)	0.73	Excluded	–
*Smoking history*				
Current or former/never	1.85 (0.79–4.34)	0.15	2.31 (0.92–5.83)	0.08
*Dose reduction during the treatment*				
Present/absent	Unable to calculate	–	Excluded	–
*Co-administration of pegfilgrastim*				
Present/absent	0.62 (0.17–2.32)	0.48	Excluded	–
*Dexamethasone dosage*				
8 mg/4 mg	0.23 (0.09–0.55)	0.001**	0.20 (0.08–0.49)	0.0005**
**(B)**				
*Age (years)*				
≥ 65/ < 65	0.72 (0.28–1.86)	0.50	Excluded	–
*Sex*				
Female/male	2.00 (0.18–22.64)	0.58	Excluded	–
*Staging*				
Metastatic/others	1.88 (0.52–6.77)	0.34	Excluded	–
*Prior treatment history*				
Present/absent	2.25 (0.95–5.33)	0.07	2.47 (1.00–6.10)	0.049*
*Hormonal receptors*				
ER, PR-positive or both/negative	1.26 (0.62–2.55)	0.52	Excluded	–
*HER2 overexpression*				
Positive/negative	1.10 (0.52–2.32)	0.80	Excluded	–
*Lymph node dissection*				
Present/absent	0.51 (0.20–1.32)	0.16	0.52 (0.19–1.39)	0.19
*BSA (m*^*2*^*)*				
≥ 1.5/ < 1.5	1.50 (0.71–3.18)	0.28	Excluded	–
*BMI (kg/m*^*2*^*)*				
≥ 25/ < 25	1.27 (0.60–2.69)	0.53	Excluded	–
*Liver dysfunction*				
Present/absent	1.18 (0.57–2.46)	0.66	Excluded	–
*Hypoalbuminemia*				
Present/absent	0.66 (0.33–1.33)	0.24	Excluded	–
*Alcohol intake (*≥ *5 days in a week)*				
Yes/no	1.48 (0.55–3.98)	0.43	Excluded	–
*Smoking history*				
Current/former or never	1.25 (0.62–2.53)	0.53	Excluded	–
*Dose reduction during the treatment*				
Present/absent	0.66 (0.18–2.45)	0.53	Excluded	–
*Co-administration of pegfilgrastim*				
Present/absent	0.60 (0.22–1.67)	0.33	Excluded	–
*Dexamethasone dosage*				
8 mg/4 mg	0.37 (0.18–0.78)	0.009**	0.35 (0.16–0.77)	0.009**

*P < 0.05, **P < 0.01.

Liver dysfunction: Grade 1 or higher aspartate aminotransferase, alanine aminotransferase, and total bilirubin levels.

The cut-off serum albumin levels is 4.1 g/dL at our facility.

*ER* estrogen receptor, *PR* progesterone receptor, *HER2* human epidermal growth factor receptor 2, *BSA* body surface area, *BMI* body mass index.

### Adverse effects related to DEX dosage

The results of DEX-dosage associated adverse effects are shown in Table 3. No patient developed pneumocystis pneumonia (PCP). The development of febrile neutropenia (FN), nausea, anorexia, fatigue, and insomnia was not different between both groups. Additionally, there were no patients experiencing grade 3/4 symptoms, except FN.

**Table 3:** Comparison of DEX dosage-related adverse effects.

	All-patient analysis			Propensity-matched analysis		
	4 mg group (n = 48)	8 mg group (n = 77)	*P* value	4 mg group (n = 39)	8 mg group (n = 39)	*P* value
**FN**						
Grade 3	27.1%	28.6%	1.00	23.1%	28.2%	0.80
**Nausea**						
Grade 1/2	31.3%	26.0%	0.54	30.8%	28.2%	1.00
**Anorexia**						
Grade ½	29.2%	37.8%	0.69	28.2%	30.8%	1.00
**Fatigue**						
Grade 1/2	35.4%	50.7%	0.10	38.5%	43.6%	0.82
**Insomnia**						
Grade 1/2	38.3%	41.6%	0.85	39.5%	43.6%	0.82

*FN* febrile neutropenia.

## Discussion

As breast cancer chemotherapy is usually administered in an outpatient setting, it is important to manage chemotherapy-induced adverse effects. Docetaxel-induced fluid retention is one of the most QOL-reducing adverse effects caused by docetaxel. A worsening of postoperative edema limits ADL in patients and adversely affects their mental health^5^. Corticosteroid is an evidence-based DIFR prophylaxis, although its appropriate administration remains unclear. Consequently, we evaluated the dose-dependent DIFR-attenuating effects of DEX.

As a result, the incidence of grade ≥ 2 and all-grade DIFR was significantly reduced by 8 mg of DEX on days 2–4 compared to 4 mg. Moreover, body weight gain after docetaxel administration was significantly decreased in the 8 mg group. Additionally, 8 mg DEX administration was identified as an independent factor for DIFR reduction. To our knowledge, this is the first study to show that high-dose DEX prophylaxis attenuates DIFR. In contrast, previous reports suggested the possibility of reducing DEX dosage in docetaxel-containing treatment^10–12^. However, docetaxel and DEX administration methods (particularly DEX initiation date and administration duration) in the present study were different from those of previous studies. Specifically, docetaxel dose for breast cancer treatment in a prospective phase I study (100 mg/m^2^) was higher than ours although their incidence of all-grade DIFR was lower^10^. A previous study reported that Japanese patients are more susceptible to docetaxel-induced toxicities^13^. Actually, maximum dosage of docetaxel in Japan is set at 75 mg/m^2^ owing to severe adverse effects^13^. They speculated that this could be induced by unknown genetic factors, higher sensitivity to adverse effects, or differences in unbound docetaxel concentrations^13^. We consider that multiple factors, such as differences in single nucleotide polymorphism in transporters and/or cytochrome P450 and higher sensitivity to the adverse effects in the Japanese population, in addition to differences of administration methods, may have affected the differences. Consequently, the results of the present study should be interpreted considering this possibility.

In addition, the mechanism(s) of its DIFR preventive efficacy remains unclear, although capillary hyperpermeability is considered as the major mechanism of DIFR^6,7^; because edema is an early inflammatory event, the potent anti-inflammatory effect of DEX may have attenuated DIFR. Moreover, the appropriate duration of prophylactic DEX also remains unclear. Therefore, further evaluations regarding its mechanisms and administration methods in larger population are required.

We also evaluated the incidence of DEX-associated adverse effects, such as gastrointestinal symptoms, fatigue, insomnia, FN, and PCP infection, resulting in non-difference. Interestingly, the incidence of facial edema was not different between the groups, although edema in the hands and feet was significantly attenuated by an increase in the dose of DEX. Because corticosteroids induce moon face^14^, it might have affected the results. In contrast, we were unable to assess loss in bone density and blood sugar elevation, which could be clinical problems in longer treatment periods, as this was a relatively short-term evaluation study. Because high-dose corticosteroids can induce stronger adverse effects, further assessment in longer evaluation periods is needed.

Risk factors for DIFR have not been reported. In this study, prior treatment history was identified as an independent risk factor for all-grade DIFR development. Most of the prior treatments in this study were perioperative anthracycline-cyclophosphamide regimens. These regimens also induce edema^15,16^; therefore, we hypothesize that existing modification on capillary hyperpermeability by prior treatment might have affected the results even though patients with baseline edema were excluded. Consequently, we should carefully monitor and educate patients with prior treatments for early and appropriate DIFR management. In contrast, DIFR is reported to be accumulative^6^, and patients in the 8 mg group tended to receive more dose reduction in the all-patient population, which could have influenced the present results. The relative dose intensity during four cycles in dose-reduced patients was 86.1 ± 5.4%. However, greater than or equal to grade 2 and all-grade DIFR were attenuated in the 8 mg group in the propensity score-matched population including a same proportion (2.6%) of dose-reduced patients for both groups. In this study, we evaluated DIFR during four cycles of the treatment, considering the perioperative setting; we conceive that dose reduction can affect the DIFR incidence in longer treatment periods, particularly cumulative docetaxel dosage > 400 mg/m^2^, which is a reported cut-off for DIFR development^5^.

Diuretics administration is the most common medication for DIFR treatment^4^. In this study, patients were prescribed furosemide 10–80 mg per day with or without daily 25–50 mg spironolactone as DIFR treatment, and 89.7% of the administered patients were manageable, suggesting that they are reliable medication. Moreover, as previously mentioned, dose reduction may also be one of the treatment strategies in long-term docetaxel treatment in a metastatic setting. Appropriate DIFR treatment, including sufficient explanation, is important in maintaining QOL besides anti-tumor efficacy. Therefore, further assessment for a proper DIFR treatment strategy is needed.

There are some limitations to the present study. First, this study was retrospectively performed with a relatively small patient population from a single institution. Second, we evaluated adverse effects by referring to a treatment diary, which almost all the patients wrote, and patients’ complaints on the treatment day. Therefore, we were unable to assess the exact date of the appearance and degradation of DIFR in some patients. Additionally, body weight data were obtained on the visiting day alone. Third, as previously described, because DIFR is accumulative, docetaxel dosage can affect its incidence and severity, suggesting that 75 mg/m^2^ docetaxel for four cycles induces less DIFR compared to higher dosage or longer administration periods. Ohsumi et al. reported that eight cycles of docetaxel treatment induce more DIFR compared to four cycles of docetaxel after four cycles of anthracycline-cyclophosphamide regimens^5^. Additionally, in this study, 10 patients received reduced treatment due to adverse effects, such as infection or taxane-associated acute pain syndrome. Consequently, it is necessary to conduct a large-scale, randomized, prospective, multicenter study in longer evaluation periods, along with careful assessment regarding dose reduction due to other adverse effects. Fourth, Kato et al. reported that DEX administration from one day before docetaxel administration can attenuate DIFR compared to its administration after a docetaxel dose^17^. Additionally, an adequate DEX administration period remains unclear. Further studies regarding DEX administration methods are needed. Finally, in the all-patient population, patients in the 8 mg group were significantly older and in a more advanced stage with lower CCr, although they were not associated with DIFR development and higher DEX dosage also exhibited better outcomes in the propensity-matched population, which adjusted the balance of the patient characteristics. Therefore, evaluation with a well-balanced population with an appropriate patient number will provide more precise results. Consequently, our preliminary findings should be confirmed in future research.

In conclusion, our study revealed that high dose of DEX prevents DIFR. Further studies are required because progress in DIFR management can significantly contribute to less onerous chemotherapy provision.

## Methods

### Patients

Patients with breast cancer who received docetaxel (75 mg/m^2^)-containing regimens between June 2015 and March 2022 were enrolled in this preliminary retrospective study. Baseline inclusion criteria were (1) age ≥ 20 years; (2) 0–1 ECOG-PS; (3) sufficient liver or renal function for treatment induction; and (4) sufficient medical records. Patients who had obvious baseline edema owing to lymph node dissection or prior treatment, were regularly administered corticosteroids and diuretics at baseline, and were not able to complete four treatment cycles were excluded. The patients were divided into two groups: 8 mg group, including patients receiving 8 mg of DEX orally on days 2–4 between July 2017 and March 2022, and 4 mg group, with those who were administered 4 mg of oral DEX on days 2–4 between June 2015 and June 2018. We hypothesized that the incidence of greater than or equal to grade 2 DIFR would be 40% in the 4 mg group and 15–20% in the 8 mg group, with a patient ratio of 2:3. To achieve 80% power with an alpha error of 5%, the required sample size was 46–75 patients in the 4 mg group, and 69–112 patients in the 8 mg group. Forty-eight patients in the 4 mg group and 77 in the 8 mg group with eligibility were analyzed.

The present study was approved by the Ethical Review Board for Life Science and Medical Research of Hokkaido University Hospital (approval number: 022-0182) and was performed in accordance with the Declaration of Helsinki and STROBE statement. In view of the retrospective nature of the study, informed consent from the participants was waived by the Ethical Review Board for Life Science and Medical Research of Hokkaido University Hospital.

### Treatment methods

Docetaxel 75 mg/m^2^ was intravenously administered for 1 h, every 3 weeks. Trastuzumab (8 mg/kg at first administration and 6 mg/kg at subsequent administration) ± pertuzumab (840 mg at first administration and 420 mg at subsequent administration) were co-administered in patients with HER2 over-expressed breast cancer. Dexamethasone 6.6 mg in the 4 mg group or 9.9 mg in the 8 mg group and granisetron 3 mg were intravenously administered to patients receiving docetaxel + cyclophosphamide 600 mg/m^2^ (TC), and intravenous DEX 6.6 mg was administered in other docetaxel-containing regimens as premedication by reference to current national antiemetic guidelines^18^. Furthermore, all the patients were administered 4–8 mg of DEX orally on days 2–4, as described previously. Diuretics such as furosemide and/or spironolactone were administered regularly or as needed to ameliorate DIFR at the physician’s discretion.

### Survey of the incidence and severity of DIFR

All required information was obtained from the patient’s medical records. The incidence of edema during the four cycles of docetaxel treatment was retrospectively evaluated in accordance with the section of localized edema in Common Terminology Criteria for Adverse Events version 5.0. We defined the evaluation periods as perioperative chemotherapy, of which approximately 90% of the participants in this study met, is usually conducted for four cycles^1^. In the present study, the primary endpoint was the comparison of greater than or equal to grade 2 DIFR incidence between the groups, as patients with greater than or equal to grade 2 symptoms definitely need medication. Secondary endpoints were configured for the assessment of all-grade DIFR incidence, variation of body weight, onset time of DIFR, risk factor(s) for the incidence of DIFR, and DEX-dose-related safety. Furthermore, propensity score-matching was performed to adjust patients’ factors between the two groups, and matched data were additionally analyzed to confirm the robustness of all-patient population results.

### Statistical analysis

The differences in patient backgrounds between the 4 and 8 mg groups were evaluated using Fisher’s exact probability test for the categorical outcome variables and the Mann–Whitney *U* test for the continuous parameters. The incidence of DIFR or other adverse effects was compared using Fisher’s exact probability method. Variation in body weight was compared using the student t-test. The cumulative incidence of DIFR was described using the Kaplan–Meier method with the log-rank test. The univariate and multivariate logistic regression analyses were carried out to find the independent risk factor(s) for the incidence of greater than or equal to grade 2 and all-grade DIFR, using the following covariates: age, sex, staging, prior treatment history, hormonal receptors expression, HER2 over-expression, lymph node dissection, BSA, BMI, liver dysfunction, hypoalbuminemia, alcohol intake, smoking history at baseline, dose reduction of docetaxel during four cycles, co-administration of pegfilgrastim, and DEX dosage. Variables that demonstrated potential associations with the incidence in univariate analysis (*P* < 0.20) were considered when building the multivariable model. Propensity score-matching was performed using the following variables: age, sex, staging, prior treatment history, hormonal receptors expression, HER2 over-expression, BSA, BMI, liver dysfunction, CCr, serum albumin, alcohol intake, smoking history, co-administration of pegfilgrastim, and dose reduction during treatment. To reduce bias with these potential confounding factors, 1:1 matching (without replacement) in the two groups was performed using the nearest neighbor method with a 0.20-width caliper of standard deviation of the logit of propensity scores.

All the analyses were performed using JMP version 16.2 statistical software (SAS Institute Japan, Tokyo, Japan). Differences were considered statistically significant when *P*-values were < 0.05.

### Ethical approval and consent to participate

All procedures performed in this study were carried out in accordance with the ethical standards of the institutional and/or national research committee and the 1964 Helsinki declaration and its later amendments or comparable ethical standards. The study was approved by the Ethical Review Board for Life Science and Medical Research of Hokkaido University Hospital (approved number: 022-0182). Given the retrospective nature of the study, informed consent from the subjects was waived by the Ethical Review Board for Life Science and Medical Research of Hokkaido University Hospital.

## Data Availability

The datasets used and/or analyzed during the current study are available from the corresponding author upon reasonable request.
